# Schistosomiasis mansoni distribution: a spatial analysis, state of Rio de Janeiro, Brazil, 2011-2023

**DOI:** 10.1590/S2237-96222026v35e20250812.en

**Published:** 2026-05-18

**Authors:** Brenno Mathias Corrêa, Alice Nardoni Marteli, Cleber Nascimento do Carmo, Daniel Savignon Marinho, Clélia Christina Mello-Silva

**Affiliations:** 1 Fundação Oswaldo Cruz, Escola Nacional de Saúde Pública Sergio Arouca, Rio de Janeiro, RJ, Brazil Fundação Oswaldo Cruz Escola Nacional de Saúde Pública Sergio Arouca Rio de Janeiro RJ Brazil; 2 Universidade Federal do Rio Grande do Sul, Programa de Pós-Graduação em Sensoriamento Remoto, Porto Alegre, RS, Brazil Universidade Federal do Rio Grande do Sul Programa de Pós-Graduação em Sensoriamento Remoto Porto Alegre RS Brazil; 3 Fundação Oswaldo Cruz, Instituto Oswaldo Cruz, Rio de Janeiro, RJ, Brazil Fundação Oswaldo Cruz Instituto Oswaldo Cruz Rio de Janeiro RJ Brazil

**Keywords:** Schistosomiasis Mansoni, *Schistosoma Mansoni*, Epidemiology, Spatial Analysis, Ecological Studies., Esquistosomiasis Mansoni, *Schistosoma mansoni*, Epidemiología, Análisis Espacial, Estudios Ecológicos.

## Abstract

**Objective:**

To analyze the spatial distribution of schistosomiasis mansoni in the municipalities of the state of Rio de Janeiro between 2011 and 2023.

**Methods:**

Exploratory spatial analysis of confirmed schistosomiasis cases recorded on the Notifiable Health Conditions Information System. Incidence rates were calculated based on annual population estimates. Correlated variables included Brazilian Institute of Geography and Statistics data on the Municipal Human Development Index, the Gini Index, and occupied households with access to piped water and sanitation, as well as the Institute for Applied Economic Research Social Vulnerability Index. Spatial dependence was assessed using Global Moran's I (*I*) and Local Moran's I (*I*_*i*_ ).

**Results:**

Between 2011 and 2023, 1,008 cases of schistosomiasis were confirmed in the state of Rio de Janeiro. The highest number of cases was recorded in 2022, with 136 confirmations. In 2023, 97 cases were confirmed. Spatial autocorrelations were found between the Social Vulnerability Index for urban infrastructure (*I*=-0.10; p-value 0.018), the composite Municipal Human Development Index (MHDI) (*I*=-0.07; p-value 0.089), and the MHDI for education (*I*=-0.07; p-value 0.076). *I*_*i*_ revealed relevant clusters in the immediate geographic region of Itaperuna, especially in Varre-Sai.

**Conclusion:**

Persistence of schistosomiasis mansoni was observed in historically endemic municipalities, covered by the Schistosomiasis Control Program, with higher incidence rates in Porciúncula, Duas Barras and Sumidouro, these being priority areas for intervention. The set of findings showed association with socioeconomic and housing indicators and indicated active surveillance and case tracing as the main control strategies.

## Resumen

**Objetivo:**

Analizar la distribución espacial de la esquistosomiasis mansoni en los municipios del estado de Río de Janeiro entre 2011 y 2023.

**Métodos:**

Análisis espacial exploratorio de los casos confirmados de esquistosomiasis registrados en el Sistema de Información de Enfermedades de Declaración Obligatoria. Las incidencias se calcularon con base en estimaciones anuales de población. Las variables correlacionadas incluyeron el índice de desarrollo humano municipal, el índice de Gini y los hogares ocupados con acceso a agua potable y saneamiento del Instituto Brasileño de Geografía y Estadística, y el índice de vulnerabilidad social del Instituto de Investigación Económica Aplicada. La dependencia espacial se evaluó mediante el índice I de Moran global (*I*) y el índice I de Moran local (*I*_*i*_ ).

**Resultados:**

Entre 2011 y 2023, se confirmaron 1008 casos de esquistosomiasis en el estado de Río de Janeiro. El mayor número de casos se registró en 2022, con 136 confirmaciones. En 2023, se confirmaron 97 casos. Se encontraron autocorrelaciones espaciales entre el índice de vulnerabilidad social de la infraestructura urbana (*I*=-0,10; p=0,018), el índice de desarrollo humano municipal (IDHM) compuesto (*I*=-0,07; p=0,089) y el IDHM para la educación (*I*=-0,07; p=0,076). El *I*_*i*_ reveló conglomerados relevantes en la región geográfica inmediata de Itaperuna, especialmente en Varre-Sai.

**Conclusión:**

Se observó persistencia de la esquistosomiasis mansoni en municipios históricamente endémicos, parte del Programa de Control de la Esquistosomiasis, con mayor incidencia en Porciúncula, Duas Barras y Sumidouro, áreas prioritarias de intervención. Los hallazgos mostraron una asociación con indicadores socioeconómicos y de vivienda, e indicaron que la vigilancia activa y la búsqueda de casos son las principales estrategias de control.

**Ethical aspects**

EThis research used public domain anonymized databases.

## Introduction

Schistosomiasis mansoni is recognized by the United Nations as a neglected tropical disease, making it a target of global efforts for its elimination by 2030^1^. It is an acute and chronic parasitic disease caused by the *Schistosoma mansoni* trematode and involves intermediate host snails of the *Biomphalaria* genus, which makes its control complex and multifactorial^2^. The disease remains a significant challenge for Brazilian public health, especially in endemic regions, where asymptomatic individuals play a relevant role in its continued presence over time^1^^,^^2^.

Schistosomiasis is strongly associated with socioeconomic inequalities and inadequate sanitation conditions, reflecting structural vulnerabilities^1^^,^^2^^,^^3^^,^^4^. Its dynamics are influenced by multiple environmental and social factors, such as climate change, land use and occupation, agricultural practices, disordered urban expansion and degradation of water bodies, which directly affect the distribution of intermediate hosts and human exposure^3^^,^^4^^,^^5^. Interaction between the environment, parasite, snails and humans is continuously shaped by changing socio-environmental processes.

Although it has low a low case fatality ratio, length of time of infection, reinfections and absence of timely diagnosis can result in significant morbidity and mortality^2^^,^^3^^,^^5^. In Brazil, the disease occurs in 19 of the country's states, nine of which are considered endemic, with a strong concentration of the disease in rural and riverside areas, where repeated exposure to contaminated water is frequent, which favors intensification of the parasitic load, the main element for the evolution of severe forms of schistosomiasis mansoni^1^^,^^2^^,^^3^^,^^6^^,^^7^.

The Schistosomiasis Control Program decentralization process in 1993 expanded municipal responsibility, but its implementation was marked by strong heterogeneity. In the state of Rio de Janeiro, many municipalities discontinued the Program's activities over the years, often prioritizing other epidemiological demands, such as arboviruses^8^^,^^9^^,^^10^. Such discontinuity reduces case detection and compromises effective health responses^6^^,^^8^^,^^9^^,^^10^.

Despite having important urban centers and relatively high socioeconomic indicators, the state of Rio de Janeiro includes regions that still face conditions marked by socio-environmental determinants, especially in the Northwest and South-Central regions of the state^2^^,^^12^^,^^13^^,^^14^. Given a scenario of predominantly passive surveillance, it becomes essential to understand the spatial patterns of schistosomiasis in order to support targeted actions contextualized to local characteristics.

Spatial analysis is a fundamental tool for investigating schistosomiasis distribution and identifying critical areas for intervention. By integrating social, environmental and epidemiological indicators, it is possible to recognize spatial clusters, risk areas and autocorrelation patterns that would not be evidenced by traditional analyses. This study aimed to analyze the spatial distribution of schistosomiasis mansoni in the state of Rio de Janeiro between 2011 and 2023, in order to contribute to the improvement of municipal surveillance and control strategies.

## Methods

Design

This is a spatial analysis with an exploratory approach, carried out based on secondary data for each municipality in the state of Rio de Janeiro, from 2011 to 2023. This design was chosen to enable analysis of spatial distribution and its correlation with socioeconomic and housing variables.

Setting

Historically, the state of Rio de Janeiro has endemic schistosomiasis mansoni areas^2^^,^^14^. This study is situated within the context of the transformations that have occurred in surveillance policies as well as the interruption of the Schistosomiasis Control Program in the state of Rio de Janeiro since 2011, which has generated the need to understand the spatial behavior of the disease since then.

Participants

The study target population consisted of residents of municipalities in the state of Rio de Janeiro. All confirmed schistosomiasis mansoni cases recorded on the Notifiable Health Conditions Information System (*Sistema de Informação de Agravos de Notificação*, SINAN) between 2011 and 2023 were included.

Data sources 

The schistosomiasis mansoni cases analyzed relate to the period from 2011 to 2023 and were obtained from SINAN, having been made available by the Brazilian Health System Department of Information Technology (*Departamento de Informação e Informática do Sistema Único de Saúde*, DATASUS), via the Ministry of Health, whereby the data were extracted on November 18, 2025^15^.

The municipal population estimates and the Gini Index were obtained via DATASUS, from official databases derived from the Brazilian Institute of Geography and Statistics (IBGE) 2010 demographic census^16^^,^^17^.

The Municipal Human Development Index (MHDI) and its sub-indices were obtained from the publication entitled *Atlas do Desenvolvimento Humano do Brasil*^13^. The Social Vulnerability Index (SVI) and its sub-indices were obtained from the publication entitled *Atlas da Vulnerabilidade Social*, produced by the Institute for Applied Economic Research (*Instituto de Pesquisa Econômica Aplicada*)^18^, all of which were also derived from the IBGE 2010 demographic census. Housing variables (Table 6803 - Permanent occupied private dwellings, according to connection to the mains water distribution network and main form of water supply; and Table 6805 - Permanent occupied private dwellings according type of sewage disposal) were obtained directly from the IBGE automatic retrieval system, based on its 2022 demographic census^19^.

Variables 

The dependent variable or outcome was the unadjusted schistosomiasis mansoni incidence rate.

The independent or explanatory variables were: a) socioeconomic: composite MHDI, MHDI Education, MHDI Longevity, MHDI Income, Gini Index, composite SVI, SVI Urban Infrastructure, SVI Human Capital, SVI Income and Work; b) housing-related: percentage of households with piped mains water and connected to the sewerage network.

Bias

Using secondary data may involve underreporting, inconsistencies in data completion and variations in case detection capacity between municipalities, which can result in underestimation and overestimation of incidence. Efforts were made to minimize these biases through the use of multiple official sources and geographic standardization of the data. Ecological fallacy, inherent to the design used, was recognized and discussed as a limitation of the study.

Study size 

The study area comprised the 92 municipalities of the state of Rio de Janeiro, organized into 14 immediate geographic regions, with an approximate area of ​​43,750 km² and an estimated population of 16,055,174 inhabitants. The state of Rio de Janeiro is located in the Southeast region of Brazil and borders with the states of Espírito Santo, Minas Gerais and São Paulo^12^. Each municipality was taken as a spatial unit of analysis.

Statistical methods

The identifiers were linked to the vector file of the municipalities based on the IBGE municipal codes, using the SIRGAS 2000 geodetic reference. Schistosomiasis incidence rates were calculated taking the sum of confirmed cases over the period as the numerator and the sum of the resident populations each year as the denominator. The scale factor adopted was 100,000 inhabitants, and the legends were defined using the Jenks natural breaks method. These processes were performed using QGIS 3.34.13.

In order to reduce rate instability in municipalities with small populations or low number of cases, global and local empirical Bayes smoothing was applied, adjusting them, respectively, towards the average for the state and the average for contiguous municipalities^20^.

The univariate and bivariate spatial autocorrelations were performed in GeoDa 1.22.0.10, using global Moran's I (*I*) and local Moran's I (*I*_*i*_ ). Global Moran's I is a statistical calculation that summarizes the degree of autocorrelation of the entire study area, ranging from -1 to +1. Values ​​close to +1 indicate positive spatial autocorrelation, and neighboring areas tend to have similar values ​​of the variable (high-high or low-low clusters). Values ​​close to -1 indicate negative spatial autocorrelation, and neighboring areas tend to have different values ​​for the variable (high-low or low-high). Values ​​close to zero suggest that the values ​​of the variable are randomly distributed over the space^21^.

Local Moran's I provides a more detailed analysis, identifying patterns of spatial autocorrelation at the local level. Analyzing it enables areas with significant spatial dependencies to be identified. This information is fundamental for the development of public policies and interventions, as it helps to adopt models, direct resources and generate hypotheses for specific areas based on well-defined spatial patterns^22^.

With regard to variable autocorrelation, first of all a spatial weight matrix needs to be defined, which determines the degree of interaction between the observations (municipalities). It is based on the idea of ​​contiguity and can be established from geographic proximity, distance between areas or a combination of these factors^23^.

The contiguity matrix applied was a first-order queen matrix. It takes neighbors to be all polygons that share any point in common, whether a boundary or just a vertex, with only immediate neighbors being incorporated into the matrix^16^^,^^17^^,^^18^. In the Monte Carlo randomization tests, 999 permutations were performed, and a p-value≤0.050 significance level was adopted for detecting spatial dependence^24^^,^^25^^,^^26^.

## Results

Between 2011 and 2023, 1,008 schistosomiasis mansoni cases were confirmed in the state of Rio de Janeiro. The highest number of cases was recorded in 2022, namely 136 confirmed cases, of which 98 (72.0%) occurred in Porciúncula. This was followed by 110 confirmed cases (10.2%) in 2014, 107 confirmed cases (9.94%) in 2015, and 106 confirmed cases (9.85%) in 2017. Ninety-seven cases were confirmed in 2023, of which 45 (46.4%) also occurred in Porciúncula^15^.

Figure 1 shows the schistosomiasis incidence rates during the period. Porciúncula, Duas Barras and Sumidouro were the municipalities with the highest unadjusted incidence rates: 111.90, 51.61 and 31.81 per 100,000 inhabitants, respectively. These municipalities were simultaneously characterized by a high number of cases compared to the other municipalities, as well as a large proportion of autochthonous cases (greater than 93.4%), a population below 20,000 inhabitants, and a history of endemic schistosomiasis. Varre-Sai was the fourth most incident municipality, with a rate of 5.95 per 100,000 inhabitants^11^^,^^12^^,^^15^.

**Figure 1:**
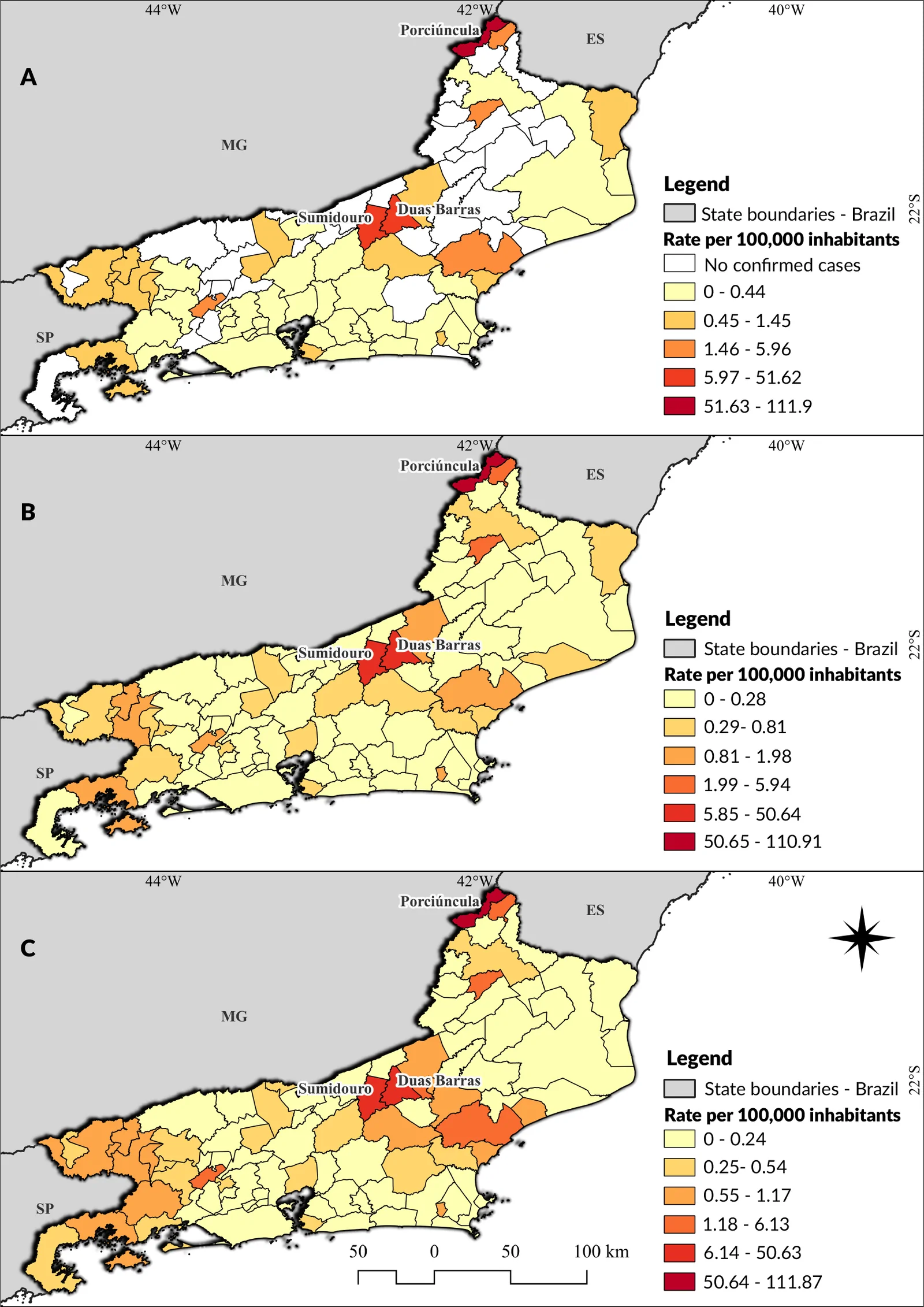
Spatial distribution of the unadjusted schistosomiasis mansoni incidence rate (A), the incidence rate smoothed using the Global Empirical Bayes method (B) and the incidence rate smoothed using the Local Empirical Bayes method (C). Rio de Janeiro, 2011-2023 (n=92)

Global univariate Moran's I analysis showed very weak positive spatial autocorrelation (*I*=0.05; p-value 0.074), which was not statistically significant. 

In the global bivariate analysis, the Social Vulnerability Index (SVI) urban infrastructure sub-index showed a statistically significant result (*I*=-0.11; p-value 0.018), which corresponds to weak negative spatial autocorrelation, whereby municipalities with better infrastructure conditions neighbor areas with high incidence rates. The opposite was also observed (Table 1).

The remaining indicators did not show significant spatial autocorrelation. Some associations were observed when statistical significance was defined at 10%: namely the composite Municipal Human Development Index (MHDI) (*I*=-0.07; p-value 0.089) and the MHDI Education sub-index (*I*=-0.07; p-value 0.076). Both indicate very weak negative spatial autocorrelations, suggesting that municipalities with higher human and educational development neighbor municipalities with low incidence rates, and that the opposite also occurs (Table 1).

In Figure 2, Maps A, B and C show the SVI Urban Infrastructure global Moran's I together with the schistosomiasis incidence rate. The maps highlight the spatial categories of the municipalities of the state of Rio de Janeiro in quadrants, which represent different spatial patterns: (A) High-Low: Araruama, Areal, Belford Roxo, Cachoeiras de Macacu, Carapebus, Campo dos Goytacazes, Cardoso Moreira, Duque de Caxias, Guapimirim, Itaboraí, Japeri, Macaé, Magé, Maricá, Mendes, Mesquita, Nilópolis, Niterói, Nova Iguaçu, Paracambi, Paty do Alferes, Petrópolis, Queimados, Quissamã, Rio de Janeiro, São Francisco de Itabapoana, São Gonçalo, São João de Meriti, Seropédica, Silva Jardim, Tanguá and Trajano de Moraes; (B) Low-Low: Angra dos Reis, Aperibé, Armação dos Búzios, Arraial do Cabo, Barra do Piraí, Barra Mansa, Bom Jesus do Itabapoana, Cabo Frio, Cambuci, Comendador Levy Gasparian, Casemiro de Abreu, Conceição de Macabu, Engenheiro Paulo de Frontin, Iguaba Grande, Itaguaí, Italva, Itaocara, Itaperuna, Itatiaia, Laje do Muriaé, Macuco, Mangaratiba, Miguel Pereira, Miracema, Paraíba do Sul, Paraty, Pinheiral, Piraí, Porto Real, Quatis, Resende, Rio Bonito, Rio Claro, Rio das Flores, Rio das Ostras, Santa Maria Madalena, Santo Antônio de Pádua, São Fidélis, São João da Barra, São José de Ubá, São Pedro da Aldeia, São Sebastião do Alto, Saquarema, Três Rios, Valência, Vassouras and Volta Redonda; (C) Low-High: Bom Jardim, Cantagalo, Carmo, Cordeiro, Duas Barras, Natividade, Nova Friburgo, Porciúncula, São José do Vale do Rio Preto, Sapucaia, Sumidouro, Teresópolis and Varre-Sai.

**Table 1:** Correlation values ​​between socioeconomic variables and the schistosomiasis incidence rate using Global Moran's I. Rio de Janeiro, 2011-2023 (n=92)

**Variables analyzed**	**Global Moran's I**	**p-value**
Composite Municipal Human Development Index (MHDI)	-0.07	0.089
MHDI Education	-0.07	0.076
MHDI Longevity	-0.04	0.199
MHDI Income	-0.04	0.179
Gini Index	-0.06	0.117
Composite Social Vulnerability Index (SVI)	-0.05	0.133
SVI Urban Infrastructure	-0.10	0.018
SVI Human Capital	0.05	0.128
SVI Income and Work	0.04	0.219
Households with piped water (%)	-0.06	0.124
Households with sewerage network connection (%)	-0.02	0.298

**Figure 2:**
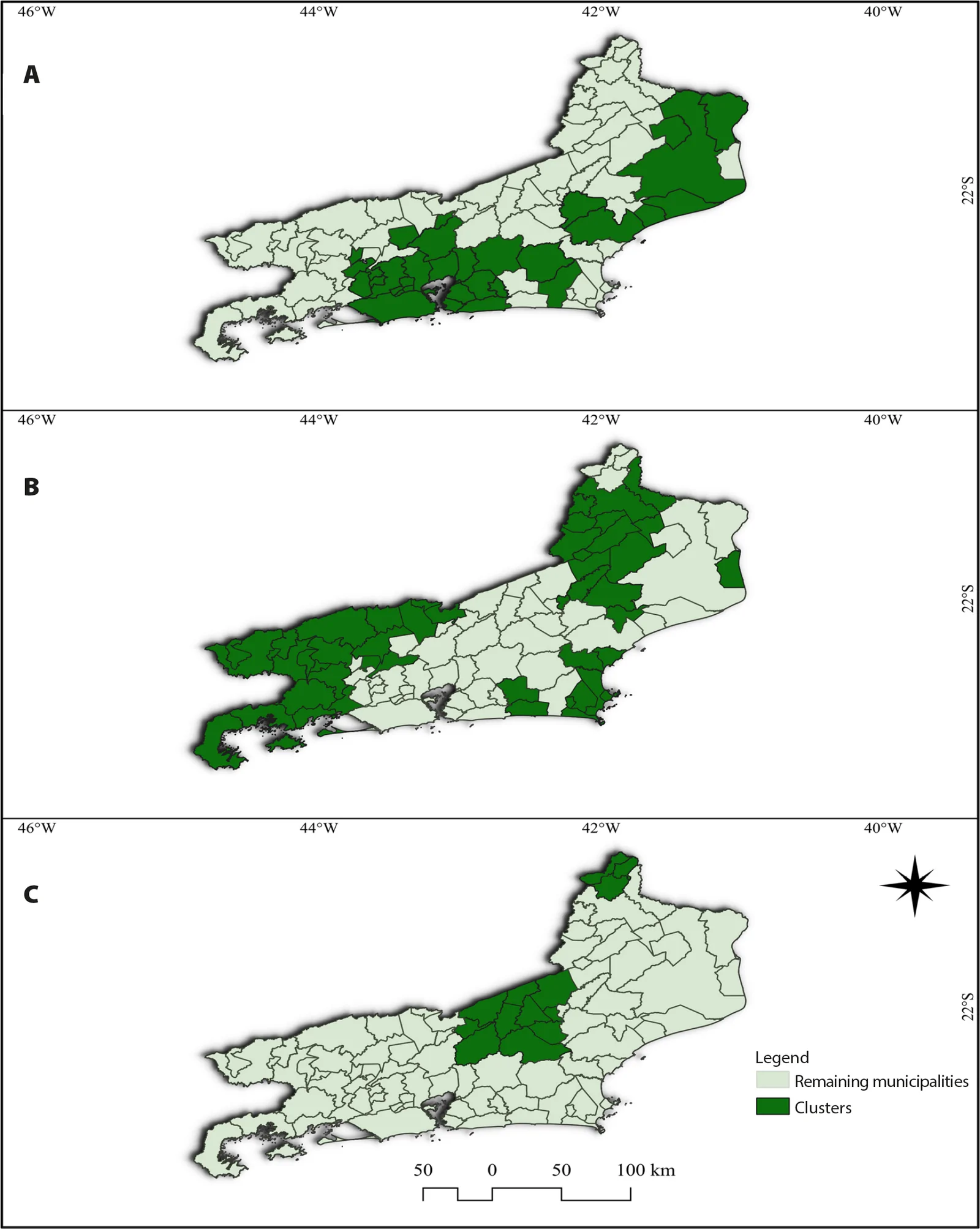
Distribution of the municipalities according to the Global bivariate Moran's I between the Social Vulnerability Index urban infrastructure sub-index and schistosomiasis mansoni incidence into low-low (A), high-low (B) and low-high (C). Rio de Janeiro, 2011-2023 (n=92)

Note: ^a^No high-high clusters were observed in this autocorrelation. 

The maps shown in Figures 3 and 4 identify the local Moran's I clusters and outliers, especially in municipalities in the immediate geographic regions of Campos dos Goytacazes, Itaperuna, Nova Friburgo, Rio de Janeiro and Santo Antônio de Pádua. The associations between socioeconomic and housing indicators and the unadjusted incidence rate are specifically shown in the municipalities of Aperibé, Natividade, São Fidélis, Santa Maria Madalena, Rio de Janeiro and Varre-Sai. The most relevant patterns were concentrated mainly in the immediate geographic region of Itaperuna.

In the univariate autocorrelation (Figure 3A), Varre-Sai showed a high-high pattern, while Natividade exhibited a low-high pattern. In the bivariate autocorrelations represented in Figure 3, maps B to E, Varre-Sai showed a low-high outlier, a pattern also observed for the longevity, education and income indicators. This means that this municipality has low human development and is contiguous to municipalities with high incidence. In the Gini Index (Figure 3F), Natividade showed a greater concentration of income in a small portion of the population, adjacent to areas of high incidence.

Regarding Figure 4, maps A, C, and D, the autocorrelations applied to the composite SVI and to the SVI Human Capital and SVI Income and Work sub-indices, showed that Varre-Sai had a high-low pattern, while Natividade had a low-high pattern. With regard to the SVI Urban Infrastructure sub-index (Figure 4B), Varre-Sai and Natividade had a low-high pattern.

Regarding the operational sanitation variables (Figures 4E and 4F), Natividade (high-high) and Varre-Sai (low-high) showed opposite behaviors. The low-low and high-low patterns were concentrated in municipalities in the immediate regions of Campos dos Goytacazes, Nova Friburgo, Rio de Janeiro and Santo Antônio de Pádua.

**Figure 3:**
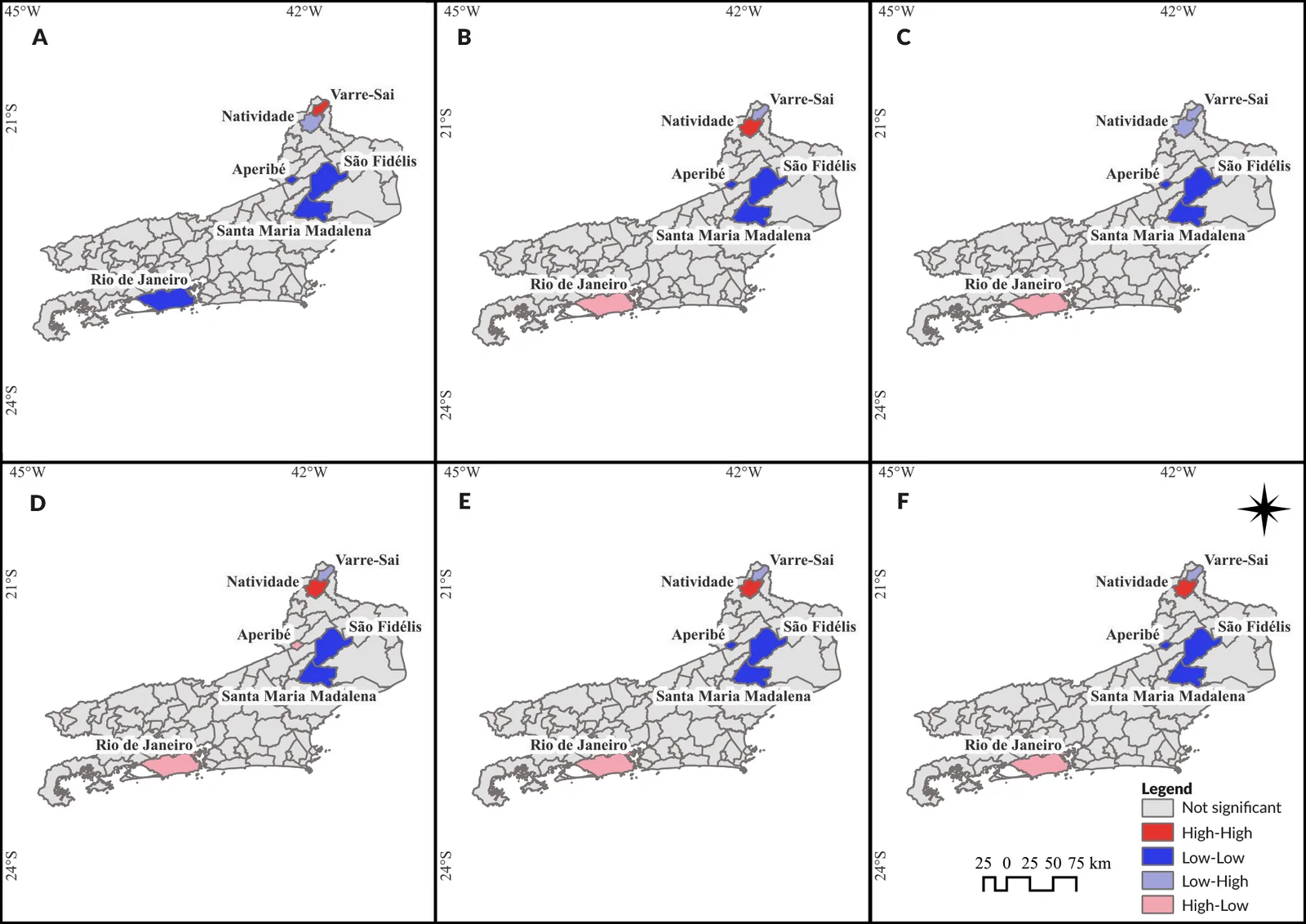
Spatial autocorrelation of the Local Moran's I for incidence and socioeconomic indicators: incidence (univariate) (A), composite Municipal Human Development Index (MHDI) (B), MHDI Longevity (C), MHDI Education (D), MHDI Income (E) and Gini Index (F). Rio de Janeiro, 2011-2023 (n=92)

**Figure 4:**
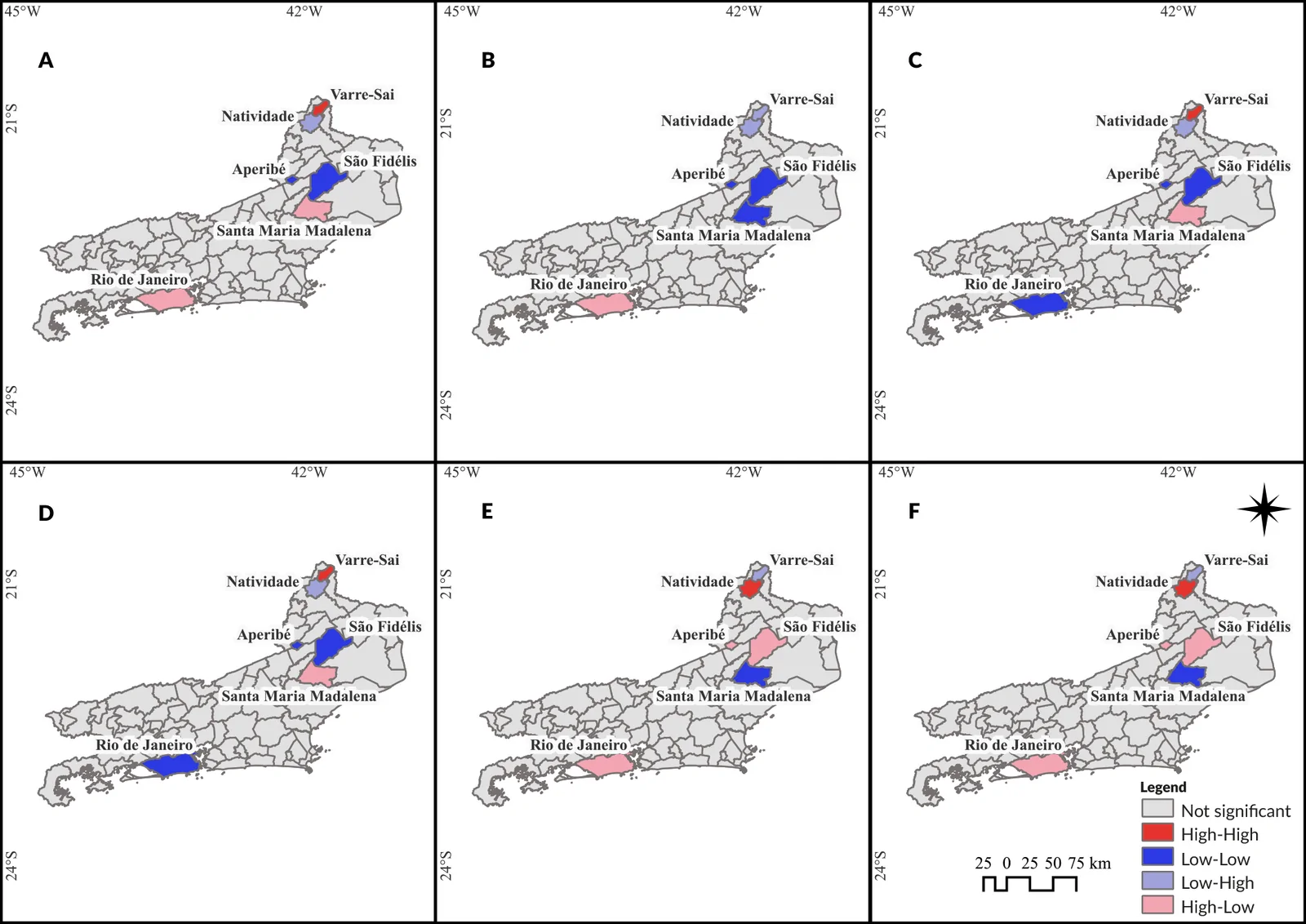
Spatial autocorrelation of Local Moran's I for socioeconomic and housing variables: composite Social Vulnerability Index (SVI) (A), SVI Urban Infrastructure (B), SVI Human Capital (C), SVI Income and Work (D), percentage households with piped mains water (E) and connected to the sewerage network (F) and schistosomiasis mansoni incidence. Rio de Janeiro, 2011-2023 (n=92)

## Discussion

The spatial analysis identified those municipalities that had the highest schistosomiasis mansoni incidence in the state of Rio de Janeiro between 2011 and 2023, highlighting Duas Barras, Porciúncula and Sumidouro as priority areas for intervention. These municipalities had already taken part in the Schistosomiasis Control Program, when positivity was 3.7% in Sumidouro (2009), 1.8% in Duas Barras (2011) and 0.9% in Porciúncula (2004), which emphasized the persistence of transmission despite the actions carried out previously^11^.

Finding spatial patterns by means of local autocorrelations enabled identification of secondary areas of concern, particularly Varre-Sai. These findings highlighted the need to resume active surveillance in the prioritized municipalities in order to prevent the re-emergence of the disease and its potential spread to neighboring localities.

Some methodological limitations should be considered when interpreting the results. The analysis was based exclusively on incidence, since there is no systematic prevalence and positivity data in the municipalities assessed^11^, which are essential for national schistosomiasis control guidelines, which use these parasitological indicators as essential parameters for stratification and definition of intervention strategies^2^^,^^3^^,^^6^^,^^8^. Absence of this information reduces the ability to fully characterize the epidemiological pattern of the disease in the state of Rio de Janeiro and may impact the accuracy of the estimates.

Reliance on secondary data is also limiting, as it implies risks of underreporting, inconsistent records and variation in diagnosis sensitivity between municipalities, which can compromise the accuracy of analyses and hinder the interpretation of transmission dynamics, especially in areas with low operational capacity. The relatively small number of confirmed cases over the 14 years analyzed may also favor the formation of spurious spatial patterns. Although Bayes smoothing was used to reduce this instability, the possibility of artificial parameters cannot be completely ruled out.

Another point to consider is that Moran's I, while robust in identifying spatial dependence, does not capture temporal variations in the disease. Added to this is the exclusion from the spatial weighting matrix of municipalities of the states of Minas Gerais, Espírito Santo and São Paulo that border with Rio de Janeiro. This may underestimate the epidemiological influence of these border areas. The analytical scope was restricted to intra-state policies and dynamics within the state of Rio de Janeiro.

The time lag due to using the Human Development Index (MHDI), Gini Index and Social Vulnerability Index (SVI) socioeconomic indicators derived from the 2010 IBGE demographic census is a further limitation, as it may not reflect recent transformations in the socioeconomic profile of the populations.

Finally, it is important to highlight the risk of ecological fallacy, common in studies with aggregated data, in which associations observed at the municipal level cannot be extrapolated to the individual level nor can causality be established.

The results of this study are consistent with the national literature on schistosomiasis and other neglected diseases. A similar pattern was identified in another study, highlighting Duas Barras, Sumidouro and Porciúncula as the municipalities with the highest incidence in the state of Rio de Janeiro between 2007 and 2017, showing a correlation between a higher number of cases and poorer sewage collection and treatment^25^. A study that investigated the persistence of schistosomiasis in the Pamparrão Valley in Sumidouro reported high sanitary vulnerability, lack of piped water and sewage treatment among the families studied, which reinforced the structuring role of socio-environmental inequalities in maintaining schistosomiasis transmission^14^.

Studies conducted in other endemic locations, such as Carne de Vaca (state of Pernambuco) and Ilha das Flores (state of Sergipe), have also shown association between schistosomiasis incidence and use of contaminated water in agriculture, absence of basic sanitation and routine exposure to water bodies^28^^,^^29^. These findings show that the behavior identified in the state of Rio de Janeiro is consistent with patterns observed in other rural and economically vulnerable areas of Brazil^28^^,^^29^^,^^30^.

This study found that Sumidouro had the worst housing and education indicators among the municipalities of the state, while Duas Barras and Varre-Sai were also characterized by low percentages of sanitation and unfavorable positions in the MHDI ranking^12^^,^^13^^,^^17^^,^^18^^,^^19^.

Porciúncula, in turn, has better housing indicators, but demonstrated high schistosomiasis incidence, which suggested that intra-municipal inequalities, spatial distribution of health services and the ecological abundance of *Biomphalaria* snails may favor transmission peaks^2^^,^^30^.

Bivariate autocorrelation of the SVI Urban Infrastructure sub-index with the state as a whole indicated that municipalities with greater infrastructural vulnerability coincided with neighbors with lower schistosomiasis incidence and vice versa. Officially, this sub-index takes three indicators into consideration: 1) percentage of people in households with water supply and sanitation, weighting 0.300; 2) percentage of the population living in households without a garbage collection service, weighting 0.300; 3) percentage of people living in households with per capita income less than half the minimum wage and who spend more than one hour commuting to work out of the total number of employed, being vulnerable people who travel daily to work, weighting 0.400^18^.

Conflicting associations between socioeconomic variables and neglected tropical disease rates have already been described in previous studies^25^. These results may be explained by the difficulty in measuring aggregated socioeconomic indicators or by factors specific to the local context, such as population density, pressure on health services and consequent underreporting in municipalities with greater infrastructural vulnerability. Proximity of the population to bodies of water contaminated by cercariae, absence of specific vector control measures in endemic areas and the weighting of the items that make up the sub-index may also influence these relationships^18^^,^^25^.

The extremely high incidence rates observed in the municipalities with the highest incidence rates may distort the state pattern, overlapping with the values ​​of other municipalities and producing inverse or seemingly paradoxical patterns in the overall bivariate assessment of the SVI Urban Infrastructure sub-index.

The results regarding the composite MHDI and the MHDI Education sub-index, however, kept to the expected pattern in epidemiological terms. The lower these indices, in the municipalities and their neighbors, the higher the incidence of schistosomiasis tends to be in these clusters, significant at 90% confidence. This contrast reflects regional inequalities and lack of equity in the allocation of public resources, which end up benefiting already developed areas while other regions lack specific structural aspects.

Local autocorrelations revealed significant clusters in the immediate region of Itaperuna. In the univariate autocorrelation, the high-high pattern in Varre-Sai suggested that shared environmental factors, geographic proximity and possible population flows may facilitate transmission. The low-high pattern observed in Natividade may indicate a phase of epidemiological transition, underreporting, or the impact of interventions. Spatial patterns observed in environmental diseases point to priority areas for epidemiological surveillance, which, in the context of schistosomiasis, suggests the need to intensify active tracing of cases in the most affected municipalities^26^.

The interpretation of the bivariate analyses in this study indicated a need for cooperation between municipalities and surveillance actions that include health education, improved living conditions, sewage collection/treatment, equitable distribution of resources, continuous monitoring of transmission hotspots and early detection of people with the disease.

Previous studies have highlighted the complexity of schistosomiasis control and the relevance of multiple simultaneous approaches that consider sociodemographic, infrastructural, behavioral, occupational factors and sensitive diagnostic methodologies^14^^,^^26^^,^^25^^,^^26^^,^^27^^,^^28^^,^^29^^,^^30^.

The findings of this study have direct implications for health policies in the municipalities of the state of Rio de Janeiro, highlighting the importance of geoprocessing and spatial statistics tools in understanding schistosomiasis distribution and in guiding public planning that considers the particularities of each region.

This study can contribute to improving the planning of schistosomiasis control strategies. Reallocation of resources from the Schistosomiasis Control Program to priority municipalities is recommended, according to the spatial patterns identified.

It is essential that future studies explore the effectiveness of interventions implemented and deepen the analyses at more detailed spatial scales, in order to identify intra-municipal inequalities and areas of greater vulnerability. Incorporating multivariate analytical models is also suggested, with a view to improving identification of socio-environmental factors associated with schistosomiasis incidence.

Point-by-point mapping of cases and analysis of their spatial relationships with sewage and snail breeding sites could also contribute to a deeper understanding of the local determinants of transmission.

## Data Availability

The database and analysis codes used in this research are available at: https://doi.org/10.48331/SCIE­LODATA.F81L1F.
