# Supplementary material for: Schistosomiasis mansoni distribution: a spatial analysis, state of Rio de Janeiro, Brazil, 2011-2023
Source: Epidemiol Serv Saude. 2026 May 18;35:e20250812. doi: 10.1590/S2237-96222026v35e20250812.en (PMC13686313; doi:10.1590/S2237-96222026v35e20250812.en)
Supplement: Translation

# Translation: Distribuição da esquistossomose mansoni: análise espacial, estado do Rio de Janeiro, 2011-2023

**Authors:** Brenno Mathias Corrêa, Alice Nardoni Marteli, Cleber Nascimento do Carmo, Daniel Savignon Marinho, Clélia Christina Mello-Silva

**Affiliations:** 1 Fundação Oswaldo Cruz, Escola Nacional de Saúde Pública Sergio Arouca, Rio de Janeiro, RJ, Brasil Fundação Oswaldo Cruz Escola Nacional de Saúde Pública Sergio Arouca Rio de Janeiro RJ Brasil; 2 Universidade Federal do Rio Grande do Sul, Programa de Pós-Graduação em Sensoriamento Remoto, Porto Alegre, RS, Brasil Universidade Federal do Rio Grande do Sul Programa de Pós-Graduação em Sensoriamento Remoto Porto Alegre RS Brasil; 3 Fundação Oswaldo Cruz, Instituto Oswaldo Cruz, Rio de Janeiro, RJ, Brasil Fundação Oswaldo Cruz Instituto Oswaldo Cruz Rio de Janeiro RJ Brasil

**Keywords:** Esquistossomose Mansoni, *Schistosoma Mansoni*, Epidemiologia, Análise Espacial, Estudos Ecológicos

## Abstract

**Objetivo:**

Analisar a distribuição espacial da esquistossomose mansoni nos municípios do estado do Rio de Janeiro entre 2011-2023.

**Métodos:**

Análise espacial exploratória dos casos confirmados de esquistossomose registrados no Sistema de Informação de Agravos de Notificação. As incidências foram calculadas com base nas estimativas populacionais anuais. As variáveis correlacionadas incluíram o índice de desenvolvimento humano municipal, o índice de Gini e os domicílios ocupados com acesso a água encanada e esgotamento sanitário do Instituto Brasileiro de Geografia e Estatística, e o índice de vulnerabilidade social do Instituto de Pesquisa Econômica Aplicada. A dependência espacial foi avaliada através do índice de Moran global (*I*) e do índice de Moran local (*I*_*i*_ ).

**Resultados:**

Entre 2011 e 2023 houve a confirmação de 1.008 casos de esquistossomose no estado do Rio de Janeiro. Em 2022, foi registrado o maior número de casos, com 136 confirmações. Em 2023, foram confirmados 97 casos. Encontraram-se autocorrelações espaciais entre o índice de vulnerabilidade social infraestrutura urbana(*I*=-0,10; p-valor 0,018), o índice de desenvolvimento humano municipal composto (*I*=-0,07; p-valor 0,089) e o educação (*I*=-0,07; p-valor 0,076). O *I*_*i*_ revelou agrupamentos relevantes na região geográfica imediata de Itaperuna, especialmente em Varre-Sai. '

**Conclusão:**

Observou-se persistência da esquistossomose mansoni em municípios historicamente endêmicos, integrantes do Programa de Controle da Esquistossomose, com maiores incidências em Porciúncula, Duas Barras e Sumidouro, áreas prioritárias de intervenção. O conjunto dos achados evidenciou associação com indicadores socioeconômicos e habitacionais e indicou vigilância ativa e busca de casos como principais estratégias de enfrentamento.

**Aspectos éticos**

Esta pesquisa utilizou bancos de dados de domínio público e anonimizados.

## Introdução

A esquistossomose mansoni é reconhecida pela Organização das Nações Unidas como uma das doenças tropicais negligenciadas, o que a tornou alvo de esforços globais para sua eliminação até 2030^1^. Ela é uma parasitose aguda e crônica causada pelo trematódeo *Schistosoma mansoni* e envolve os hospedeiros intermediários caramujos do gênero *Biomphalaria*, o que torna o controle complexo e multicausal^2^. A doença permanece como um desafio significativo para a saúde pública brasileira, especialmente em regiões endêmicas, nas quais indivíduos assintomáticos desempenham papel relevante na sua manutenção ao longo do tempo^1^^,^^2^.

A esquistossomose apresenta forte relação com desigualdades socioeconômicas e condições inadequadas de saneamento, o que reflete vulnerabilidades estruturais^1^^,^^2^^,^^3^^,^^4^. Sua dinâmica é influenciada por múltiplos fatores ambientais e sociais, como mudanças climáticas, uso e ocupação do solo, práticas agrícolas, expansão urbana desordenada e degradação de corpos hídricos, que afetam diretamente a distribuição dos hospedeiros intermediários e a exposição humana^3^^,^^4^^,^^5^. A interação entre ambiente, parasita, caramujos e seres humanos é continuamente moldada por processos socioambientais em transformação.

Embora apresente baixa letalidade, o tempo de infecção, as reinfecções e a ausência de diagnóstico oportuno podem resultar em morbimortalidade acentuada^2^^,^^3^^,^^5^. No Brasil, a doença ocorre em 19 estados, dos quais nove são considerados endêmicos, com forte concentração da doença em áreas rurais e ribeirinhas, onde a exposição repetida a águas contaminadas é frequente, o que favorece a intensificação da carga parasitária, principal elemento para a evolução de formas graves da esquistossomose mansoni^1^^,^^2^^,^^3^^,^^6^^,^^7^.

O processo de descentralização do Programa de Controle da Esquistossomose, em 1993, ampliou a responsabilidade municipal, mas sua implementação foi marcada por forte heterogeneidade. No estado do Rio de Janeiro, muitos municípios descontinuaram as atividades do programa ao longo dos anos, frequentemente com priorização de outras demandas epidemiológicas, como as arboviroses^8^^,^^9^^,^^10^. Essa descontinuidade reduz a detecção de casos e compromete respostas sanitárias resolutivas^6^^,^^8^^,^^9^^,^^10^. 

Apesar de apresentar importantes centros urbanos e indicadores socioeconômicos relativamente elevados, o estado do Rio de Janeiro inclui regiões que ainda enfrentam condições marcadas por determinantes socioambientais, especialmente no Noroeste e no Centro-Sul do estado^2^^,^^12^^,^^13^^,^^14^. Diante de um cenário de vigilância predominantemente passiva, torna-se essencial compreender os padrões espaciais da esquistossomose para subsidiar ações direcionadas e contextualizadas às características locais. 

A análise espacial constitui uma ferramenta fundamental para investigar a distribuição da esquistossomose e identificar áreas críticas para intervenção. Ao integrar indicadores sociais, ambientais e epidemiológicos, foi possível reconhecer agrupamentos espaciais, áreas de risco e padrões de autocorrelação que não seriam evidenciados pelas análises tradicionais. Este estudo teve por objetivo analisar a distribuição espacial da esquistossomose mansoni no estado do Rio de Janeiro entre 2011 e 2023, de forma a contribuir para o aprimoramento das estratégias municipais de vigilância e de controle.

## Métodos

Delineamento do estudo

Trata-se de análise espacial com abordagem exploratória, realizada com base em dados secundários por município do estado do Rio de Janeiro, no período de 2011 a 2023. Esse delineamento foi escolhido para permitir analisar a distribuição espacial e correlacioná-la às variáveis socioeconômicas e habitacionais.

Contexto

Historicamente, o estado do Rio de Janeiro possui áreas endêmicas para esquistossomose mansoni^2^^,^^14^. Este estudo insere-se no contexto das transformações ocorridas nas políticas de vigilância e da interrupção do Programa de Controle da Esquistossomose no território fluminense desde 2011, o que gerou a necessidade de compreender o comportamento espacial da doença nesse período recente.

Participantes

A população-alvo do estudo foi composta pelos residentes dos municípios do estado do Rio de Janeiro. Incluíram-se todos os casos confirmados de esquistossomose mansoni registrados no Sistema de Informação de Agravos de Notificação (Sinan) entre 2011 e 2023.

Fontes de dados

Os casos de esquistossomose mansoni analisados foram referentes ao período de 2011 a 2023 e foram obtidos do Sinan, disponibilizado pelo Departamento de Informação e Informática do Sistema Único de Saúde (DATASUS), do Ministério da Saúde, com dados extraídos em 18 de novembro de 2025^15^.

As estimativas populacionais municipais e o índice de Gini foram obtidos por meio do DATASUS, a partir de bases oficiais derivadas do censo demográfico de 2010 do Instituto Brasileiro de Geografia e Estatística (IBGE)^16^^,^^17^.

O índice de desenvolvimento humano municipal (IDHM) e seus subíndices foram obtidos do *Atlas do Desenvolvimento Humano do Brasil*^13^. O índice de vulnerabilidade social (IVS) e seus subíndices foram obtidos do *Atlas da Vulnerabilidade Social* do Instituto de Pesquisa Econômica Aplicada^18^. Todos também derivados do censo demográfico de 2010 do IBGE. As variáveis habitacionais (Tabela 6803 - Domicílios particulares permanentes ocupados por existência de ligação à rede geral de distribuição de água e principal forma de abastecimento de água; e Tabela 6805 - Domicílios particulares permanentes ocupados por tipo de esgotamento sanitário) foram obtidas diretamente do sistema IBGE de recuperação automática, referente ao censo demográfico de 2022^19^.

Variáveis

A variável dependente ou desfecho foi a taxa bruta de incidência de esquistossomose mansoni.

As variáveis independentes ou explicativas foram: socioeconômicas: IDHM composto, IDHM educação, IDHM longevidade, IDHM renda, índice de Gini, IVS composto, IVS infraestrutura urbana, IVS capital humano, IVS renda e trabalho; habitacionais: percentual de domicílios com acesso à rede geral de água encanada e de esgotamento sanitário.

Viés

O uso de dados secundários pode envolver subnotificação, inconsistências de preenchimento e variações na capacidade de detecção de casos entre os municípios, o que pode resultar em subestimação e em superestimação da incidência. Buscou-se minimizar esses vieses por meio da utilização de múltiplas fontes oficiais e da padronização geográfica dos dados. A falácia ecológica, inerente ao delineamento adotado, foi reconhecida e discutida como limitação do estudo.

Tamanho do estudo

A área de estudo compreende os 92 municípios do estado do Rio de Janeiro, organizados em 14 regiões geográficas imediatas, com área aproximada de 43.750 km² e população estimada em 16.055.174 habitantes. O estado localiza-se na região Sudeste do Brasil e faz fronteira com os estados do Espírito Santo, de Minas Gerais e de São Paulo^12^. Cada município foi adotado como unidade espacial de análise.

Métodos estatísticos

Os identificadores foram associados ao arquivo vetorial dos municípios com base no código municipal do IBGE, com o referencial geodésico SIRGAS 2000. As taxas de incidência da esquistossomose foram calculadas com a consideração do somatório dos casos confirmados ao longo do período como numerador e do somatório das populações residentes a cada ano como denominador. O fator de escala adotado foi de 100 mil habitantes, as legendas foram definidas pelo método de quebras naturais de Jenks. Esses processos foram realizados no programa QGIS 3.34.13.

Para reduzir a instabilidade das taxas em municípios com pequenas populações ou baixo número de casos, aplicaram-se as suavizações bayesianas empíricas global e local, ajustando-as, respectivamente, em direção à média estadual e à média dos municípios contíguos^20^.

No programa GeoDa 1.22.0.10, foram realizadas as autocorrelações espaciais univariada e bivariada pelo índice de Moran global (*I*) e local (*I*_*i*_ ). O índice de Moran global é um cálculo estatístico que resume o grau de autocorrelação de toda a área de estudo, com variação de -1 a +1. Valores próximos a +1 indicam uma autocorrelação espacial positiva e áreas próximas tendem a ter valores similares da variável (agrupamentos de alto-alto ou baixo-baixo). Valores próximos a -1 indicam uma autocorrelação espacial negativa e áreas próximas tendem a ter valores diferentes da variável (alto-baixo ou baixo-alto). Valores próximos a zero sugerem que os valores da variável são distribuídos aleatoriamente pelo espaço^21^.

O índice de Moran local proporciona uma análise mais detalhada, com identificação dos padrões de autocorrelação espacial em nível local. Ao analisá-lo, identificam-se áreas com dependências espaciais significativas. São informações fundamentais para a elaboração de políticas públicas e de intervenções, pois ajudam a adotar modelos, direcionar recursos e gerar hipóteses para áreas específicas com base em padrões espaciais bem definidos^22^.

Para a autocorrelação das variáveis, é necessário primeiramente definir uma matriz de pesos espaciais, a qual determina o grau de interação entre as observações (municípios). Ela baseia-se na ideia de contiguidade e pode ser estabelecida a partir da vizinhança geográfica, da distância entre as áreas ou da combinação desses fatores^23^.

A matriz de contiguidade aplicada foi a do tipo *queen* de primeira ordem. Ela considera como vizinhos todos os polígonos que compartilham qualquer ponto em comum. Seja uma fronteira ou apenas um vértice, com incorporação na matriz apenas dos vizinhos imediatos^16^^,^^17^^,^^18^. Nos testes de randomização de Monte Carlo, foram realizadas 999 permutações e foi adotado o nível de significância de p-valor≤0,050 para detecção de dependência espacial^24^^,^^25^^,^^26^.

## Resultados

Entre 2011 e 2023, houve a confirmação de 1.008 casos de esquistossomose mansoni no estado do Rio de Janeiro. Em 2022, foi registrado o maior número de casos, 136 confirmados, dos quais 98 (72,0%) ocorreram em Porciúncula. Seguido dos anos de 2014, com 110 casos confirmados (10,2%), 2015, com 107 casos confirmados (9,94%), e 2017, com 106 casos confirmados (9,85%). Em 2023, 97 casos foram confirmados, dos quais 45 (46,4%) ocorreram também em Porciúncula^15^.

A Figura 1 exibiu as taxas de incidência da esquistossomose no período. Porciúncula, Duas Barras e Sumidouro apresentaram as maiores taxas brutas de incidência: 111,90, 51,61 e 31,81 por 100 mil habitantes. Esses municípios foram caracterizados simultaneamente por elevado número de casos em relação aos outros municípios, ampla proporção de casos autóctones (acima de 93,4%), população abaixo de 20 mil habitantes e histórico endêmico. Varre-Sai foi o quarto município mais incidente, com taxa de 5,95 por 100 mil habitantes^11^^,^^12^^,^^15^.

**Figura 1.** Distribuição espacial da taxa bruta de incidência (A), da taxa suavizada pelo método bayesiano empírico global (B) e da taxa suavizada pelo método bayesiano empírico local (C) de esquistossomose mansoni. Rio de Janeiro, 2011-2023 (n=92)

A análise univariada do índice de Moran global apresentou autocorrelação espacial positiva muito fraca (*I*= 0,05; p-valor 0,074), não estatisticamente significativa. 

Na análise bivariada global, o índice de vulnerabilidade social (IVS) infraestrutura urbana foi o indicador que apresentou resultado estatisticamente significativo (*I*=-0,11; p-valor 0,018), o que corresponde a autocorrelação espacial negativa fraca, na qual municípios com melhores condições de infraestrutura estão próximos de áreas com altas taxas de incidência. O inverso também foi observado (Tabela 1).

Os demais indicadores não apresentaram autocorrelação espacial significativa. Observaram-se algumas associações com significância estatística ao nível de 10%: o índice de desenvolvimento humano municipal (IDHM) composto (*I*=-0,07; p-valor 0,089) e o IDHM educação (*I*=-0,07; p-valor 0,076). Ambas indicam autocorrelações espaciais negativas muito fracas, o que sugere que municípios com maior desenvolvimento humano e educacional apresentam vizinhança com baixas taxas de incidência e que o oposto igualmente ocorre (Tabela 1).

A Figura 2, em seus mapas A, B e C, mostrou o índice de Moran global do IVS infraestrutura urbana com a taxa de incidência da esquistossomose. Os mapas destacaram as categorias espaciais dos municípios do estado Rio de Janeiro em quadrantes, que representam diferentes padrões espaciais: (A) alto-baixo: Araruama, Areal, Belford Roxo, Cachoeiras de Macacu, Carapebus, Campo dos Goytacazes, Cardoso Moreira, Duque de Caxias, Guapimirim, Itaboraí, Japeri, Macaé, Magé, Maricá, Mendes, Mesquita, Nilópolis, Niterói, Nova Iguaçu, Paracambi, Paty do Alferes, Petrópolis, Queimados, Quissamã, Rio de Janeiro, São Francisco de Itabapoana, São Gonçalo, São João de Meriti, Seropédica, Silva Jardim, Tanguá e Trajano de Moraes; (B) baixo-Baixo: Angra dos Reis, Aperibé, Armação dos Búzios, Arraial do Cabo, Barra do Piraí, Barra Mansa, Bom Jesus do Itabapoana, Cabo Frio, Cambuci, Comendador Levy Gasparian, Casemiro de Abreu, Conceição de Macabu, Engenheiro Paulo de Frontin, Iguaba Grande, Itaguaí, Italva, Itaocara, Itaperuna, Itatiaia, Laje do Muriaé, Macuco, Mangaratiba, Miguel Pereira, Miracema, Paraíba do Sul, Paraty, Pinheiral, Piraí, Porto Real, Quatis, Resende, Rio Bonito, Rio Claro, Rio das Flores, Rio das Ostras, Santa Maria Madalena, Santo Antônio de Pádua, São Fidélis, São João da Barra, São José de Ubá, São Pedro da Aldeia, São Sebastião do Alto, Saquarema, Três Rios, Valência, Vassouras e Volta Redonda; (C) baixo-alto: Bom Jardim, Cantagalo, Carmo, Cordeiro, Duas Barras, Natividade, Nova Friburgo, Porciúncula, São José do Vale do Rio Preto, Sapucaia, Sumidouro, Teresópolis e Varre-Sai.

**Tabela 1:** Valores da correlação entre variáveis socioeconômicas e a taxa de incidência de esquistossomose pelo índice de Moran global. Rio de Janeiro, 2011-2023 (n=92)

Variáveis analisadas	Índice de Moran global	p-valor
Índice de desenvolvimento humano municipal (IDHM) composto	-0,07	0,089
IDHM educação	-0,07	0,076
IDHM longevidade	-0,04	0,199
IDHM renda	-0,04	0,179
Índice de Gini	-0,06	0,117
Índice de vulnerabilidade social (IVS) composto	-0,05	0,133
IVS infraestrutura urbana	-0,10	0,018
IVS capital humano	0,05	0,128
IVS renda e trabalho	0,04	0.219
Domicílios com água encanada (%)	-0,06	0,124
Domicílios com esgotamento sanitário (%)	-0,02	0,298

**Figura 2.** Distribuição dos municípios segundo o índice Moran global bivariado entre o subíndice infraestrutura urbana do índice de vulnerabilidade social e a incidência de esquistossomose mansoni em baixo-baixo (A), alto-baixo (B) e baixo-alto (C). Rio de Janeiro, 2011-2023 (n=92)

^a^Não foram observados agrupamentos do tipo alto-alto nessa autocorrelação.

Os mapas representados nas Figuras 3 e 4 identificam os agrupamentos e os pontos atípicos no índice de Moran local, sobretudo em municípios das regiões geográficas imediatas de Campos dos Goytacazes, Itaperuna, Nova Friburgo, Rio de Janeiro e Santo Antônio de Pádua. As associações dos indicadores socioeconômicos e habitacionais com a taxa bruta incidência são exibidas especificamente nos municípios de Aperibé, Natividade, São Fidélis, Santa Maria Madalena, Rio de Janeiro e Varre-Sai. Os padrões mais relevantes concentraram-se principalmente na região imediata de Itaperuna.

Na autocorrelação univariada (Figura 3A), Varre-Sai apresentou padrão alto-alto, enquanto Natividade exibiu baixo-alto. Nas autocorrelações bivariadas representadas nos mapas de B a E da Figura 3, Varre-Sai apresentou ponto atípico baixo-alto, padrão também observado nos indicadores de longevidade, educação e renda. Isso quer dizer que o município possui baixo desenvolvimento humano contíguo a municípios de alta incidência. No índice de Gini (Figura 3F), Natividade apontou maior concentração de renda em parcela reduzida da população, adjacente a áreas de alta incidência.

Em relação à Figura 4, mapas A, C e D, as autocorrelações aplicadas às variáveis do IVS, IVS capital humano e IVS renda e trabalho, Varre-Sai assumiu padrão alto-baixo, enquanto Natividade exibiu baixo-alto. No IVS infraestrutura urbana (Figura 4B), Varre-Sai e Natividade apresentaram formato baixo-alto. 

Nas variáveis operacionais de saneamento (Figuras 4E e 4F), Natividade (alto-alto) e Varre-Sai (baixo-alto) manifestaram comportamentos opostos. Já os padrões baixo-baixo e alto-baixo ficaram concentrados em municípios das regiões imediatas de Campos dos Goytacazes, Nova Friburgo, Rio de Janeiro e Santo Antônio de Pádua.

**Figura 3:** Autocorrelação espacial do índice de Moran local da incidência e dos indicadores socioeconômicos: incidência (univariada) (A), índice de desenvolvimento humano municipal (IDHM) composto (B), IDHM longevidade (C), IDHM educação (D), IDHM renda (E) e índice de Gini (F). Rio de Janeiro, 2011-2023 (n=92)

**Figura 4:** Autocorrelação espacial do índice de Moran local das variáveis socioeconômicas e habitacionais: índice de vulnerabilidade social (IVS) composto (A), IVS infraestrutura urbana (B), IVS capital humano (C), IVS renda e trabalho (D), percentual de domicílios com rede geral de água encanada (E) e com rede geral de esgoto (F) e a incidência de esquistossomose mansoni. Rio de Janeiro, 2011-2023 (n=92)

Discussão

A análise espacial identificou os municípios com maior incidência de esquistossomose mansoni no estado do Rio de Janeiro entre 2011 e 2023, com destaque para Duas Barras, Porciúncula e Sumidouro como áreas prioritárias de intervenção. Esses municípios já haviam participado do Programa de Controle da Esquistossomose e apresentaram positividade de 3,7% em Sumidouro (2009), 1,8% em Duas Barras (2011) e 0,9% em Porciúncula (2004), o que reforçou a persistência da transmissão apesar das ações previamente realizadas^11^.

A constatação de padrões espaciais por meio das autocorrelações locais permitiu identificar áreas secundárias de atenção, com destaque para Varre-Sai. Esses achados evidenciaram a necessidade de retomar a vigilância ativa nos municípios priorizados, a fim de prevenir a reemergência da doença e o seu potencial espalhamento para localidades vizinhas.

Algumas limitações metodológicas devem ser consideradas na interpretação dos resultados. A análise baseou-se exclusivamente na incidência, uma vez que não há dados sistemáticos de prevalência e de positividade nos municípios avaliados^11^, os quais são essenciais para as diretrizes nacionais de controle da esquistossomose, que utilizam tais indicadores parasitológicos como parâmetros essenciais para estratificação e definição de estratégias de intervenção^2^^,^^3^^,^^6^^,^^8^. A ausência dessas informações reduz a capacidade de caracterizar plenamente o padrão epidemiológico da doença no estado e pode impactar o rigor das estimativas.

A dependência de dados secundários também é limitante, pois implica riscos de subnotificação, inconsistência de registros e variação na sensibilidade diagnóstica entre municípios, o que pode comprometer a precisão das análises e dificultar a interpretação da dinâmica de transmissão, especialmente em áreas com baixa capacidade operacional. O número relativamente reduzido de casos confirmados ao longo de 14 anos analisados também pode favorecer a formação de padrões espaciais espúrios. Embora a suavização bayesiana tenha sido utilizada para reduzir essa instabilidade, a possibilidade de parâmetros artificiais não pode ser completamente descartada.

Outro ponto a se considerar é que o índice de Moran, embora robusto para identificar dependência espacial, não capta variações temporais da doença. Soma-se a isso a não inclusão de municípios fronteiriços de Minas Gerais, do Espírito Santo e de São Paulo na matriz de pesos espaciais. Isso pode subestimar a influência epidemiológica dessas áreas limítrofes. Contudo, o escopo analítico restringiu-se às políticas e às dinâmicas intraestaduais no estado do Rio de Janeiro.

A defasagem temporal dos indicadores socioeconômicos índice de desenvolvimento humano (IDHM), índice de Gini e índice de vulnerabilidade social (IVS) do censo demográfico de 2010 do IBGE constitui mais uma limitação, pois pode não refletir transformações recentes no perfil socioeconômico das populações.

Destaca-se, por fim, o risco de falácia ecológica, comum em estudos com dados agregados, nos quais as associações observadas a nível municipal não podem ser extrapoladas ao nível individual nem estabelecer causalidade.

Os resultados deste estudo dialogam com a literatura nacional sobre esquistossomose e outros agravos negligenciados. Foi identificado padrão semelhante em outro estudo, com destaque para Duas Barras, Sumidouro e Porciúncula como os municípios mais incidentes do estado entre 2007 e 2017, com relação entre maior número de casos e pior coleta e tratamento de esgoto^25^. Já estudo que investigou a persistência da esquistossomose no Vale do Pamparrão em Sumidouro, relatou elevada vulnerabilidade sanitária e ausência de água encanada e de esgotamento entre as famílias estudadas, o que reforçou o papel estruturante das desigualdades socioambientais na manutenção da transmissão^14^.

Estudos realizados em outras localidades endêmicas, como Carne de Vaca (Pernambuco) e Ilha das Flores (Sergipe), também evidenciaram associação entre a incidência de esquistossomose e o uso de água contaminada nas práticas agrícolas, a ausência de saneamento básico e a exposição rotineira a corpos hídricos^28^^,^^29^. Esses achados mostram que o comportamento identificado no estado do Rio de Janeiro é consistente com padrões observados em áreas rurais e economicamente vulneráveis do país^28^^,^^29^^,^^30^.

Este estudo observou que Sumidouro apresentou os piores indicadores habitacionais e educacionais entre os municípios do estado, enquanto Duas Barras e Varre-Sai também se caracterizaram por baixos percentuais de esgotamento sanitário e posições desfavoráveis no ranking do IDHM^12^^,^^13^^,^^17^^,^^18^^,^^19^.

Porciúncula, por sua vez, tem melhores indicadores habitacionais, mas demonstrou elevada incidência da doença, o que sugeriu que desigualdades intramunicipais, a distribuição espacial dos serviços e a abundância ecológica de caramujos *Biomphalaria* podem favorecer picos de transmissão^2^^,^^30^.

A autocorrelação bivariada do IVS infraestrutura urbana com a consideração de todo o estado indicou que municípios com maior vulnerabilidade infraestrutural coincidiram com vizinhos de menor incidência da doença e vice-versa. Oficialmente, esse subíndice considera três indicadores: 1) percentual de pessoas em domicílios com abastecimento de água e esgotamento sanitário, peso 0,300; 2) percentual da população que vive em domicílios sem serviço de coleta de lixo, peso 0,300; 3) percentual de pessoas que vivem em domicílios com renda per capita inferior a meio salário-mínimo e que gastam mais de uma hora até o trabalho no total de pessoas ocupadas, vulneráveis e que retornam diariamente ao trabalho, peso 0,400^18^.

Associações conflitantes entre variáveis socioeconômicas e as taxas de doenças tropicais negligenciadas já foram descritas em estudos prévios^25^. Esses resultados podem ser explicados pela difícil mensuração de indicadores socioeconômicos agregados ou por fatores específicos ao contexto local, como a densidade populacional, a pressão sobre os serviços de saúde e a consequente subnotificação em municípios de maior vulnerabilidade infraestrutural. A proximidade da população a corpos d'água contaminados por cercárias, a ausência de medidas específicas de controle de vetores em áreas endêmicas e a própria ponderação dos pesos que compõem o subíndice também podem influenciar essas relações^18^^,^^25^.

As taxas de incidências extremamente elevadas observadas nos municípios mais incidentes podem distorcer o padrão estadual, com sobreposição aos valores dos demais municípios e produção de padrões inversos ou aparentemente paradoxais na avaliação bivariada global do IVS infraestrutura.

Os resultados referentes ao IDHM e ao IDHM educação, porém, mantiveram padrão esperado em termos epidemiológicos. Quanto menor esses índices, nos municípios e seus vizinhos, maior tende a ser a incidência da esquistossomose nesses agrupamentos, significativo a 90% de confiança. Esse contraste reflete as desigualdades regionais e a falta de equidade na alocação de recursos públicos, os quais acabam por beneficiar áreas já desenvolvidas enquanto outras regiões carecem de aspectos estruturais específicos.

As autocorrelações locais revelaram agrupamentos importantes na região imediata de Itaperuna. Na autocorrelação univariada, o padrão alto-alto em Varre-Sai sugeriu que fatores ambientais compartilhados, proximidade geográfica e possíveis fluxos populacionais podem facilitar a transmissão. Já o padrão baixo-alto observado em Natividade pode indicar uma fase de transição epidemiológica, subnotificação ou impacto de intervenções. Os padrões espaciais observados em doenças ambientais apontam áreas prioritárias para vigilância epidemiológica, o que, no contexto da esquistossomose, propõe a necessidade de intensificar a busca ativa de casos nos municípios mais incidentes^26^.

A interpretação das análises bivariadas deste estudo indicou a demanda de cooperação intermunicipal e de ações de vigilância que contemplem educação em saúde, melhoria das condições de vida, coleta/tratamento de esgoto, equidade na distribuição de recursos, monitoramento contínuo dos focos de transmissão e detecção precoce dos doentes.

Estudos prévios sublinharam a complexidade do controle da esquistossomose e a relevância de múltiplas abordagens simultâneas, que considerem fatores sociodemográficos, infraestruturais, comportamentais, ocupacionais e metodologias diagnósticas sensíveis^14^^,^^26^^,^^27^^,^^28^^,^^29^^,^^30^.

Os achados deste trabalho têm implicações diretas para as políticas de saúde nos municípios do estado do Rio de Janeiro, com destaque para a importância das ferramentas de geoprocessamento e estatística espacial na compreensão da distribuição da esquistossomose e na orientação de planejamento público que considere as particularidades de cada região. 

Espera-se contribuir para o aprimoramento do planejamento das estratégias de controle da esquistossomose. Recomenda-se a realocação de recursos do Programa de Controle da Esquistossomose para os municípios prioritários, de acordo com os padrões espaciais identificados.

É imprescindível que estudos futuros explorem a eficácia das intervenções implementadas e aprofundem as análises em escalas espaciais mais detalhadas, de modo a identificar desigualdades intramunicipais e áreas de maior vulnerabilidade. Sugere-se ainda a incorporação de modelos analíticos multivariados, com vistas ao aprimoramento da identificação de fatores socioambientais associados à incidência da doença.

Mapeamentos pontuais dos casos e análise das suas relações espaciais com efluentes sanitários e criadouros de caramujos também poderão contribuir para uma compreensão mais aprofundada dos determinantes locais da transmissão.

## Data Availability

O banco de dados e os códigos de análise utilizados na pesquisa estão disponíveis em: https://doi.org/10.48331/SCIELODATA.F81L1F.
